# The importance of extracellular vesicle purification for downstream analysis: A comparison of differential centrifugation and size exclusion chromatography for helminth pathogens

**DOI:** 10.1371/journal.pntd.0007191

**Published:** 2019-02-27

**Authors:** Chelsea N. Davis, Helen Phillips, John J. Tomes, Martin T. Swain, Toby J. Wilkinson, Peter M. Brophy, Russell M. Morphew

**Affiliations:** Institute of Biological, Environmental and Rural Sciences, Aberystwyth University, Aberystwyth, United Kingdom; Wellcome Trust Sanger Institute, UNITED KINGDOM

## Abstract

**Background:**

Robust protocols for the isolation of extracellular vesicles (EVs) from the rest of their excretory-secretory products are necessary for downstream studies and application development. The most widely used purification method of EVs for helminth pathogens is currently differential centrifugation (DC). In contrast, size exclusion chromatography (SEC) has been included in the purification pipeline for EVs from other pathogens, highlighting there is not an agreed research community ‘gold standard’ for EV isolation. In this case study, *Fasciola hepatica* from natural populations were cultured in order to collect EVs from culture media and evaluate a SEC or DC approach to pathogen helminth EV purification.

**Methodology/Principal findings:**

Transmission electron and atomic force microscopy demonstrated that EVs prepared by SEC were both smaller in size and less diverse than EV resolved by DC. Protein quantification and Western blotting further demonstrated that SEC purification realised a higher EV purity to free excretory-secretory protein (ESP) yield ratio compared to DC approaches as evident by the reduction of soluble free cathepsin L proteases in SEC EV preparations. Proteomic analysis further highlighted DC contamination from ESP as shown by an increased diversity of protein identifications and unique peptide hits in DC EVs as compared to SEC EVs. In addition, SEC purified EVs contained less tegumental based proteins than DC purified EVs.

**Conclusions/Significance:**

The data suggests that DC and SEC purification methods do not isolate equivalent EV population profiles and caution should be taken in the choice of EV purification utilised, with certain protocols for DC preparations including more free ES proteins and tegumental artefacts. We propose that SEC methods should be used for EV purification prior to downstream studies.

## Author summary

Recent pathogen research has identified extracellular vesicle (EV) release from many organisms. EVs are small membrane bound organelles, which have different origins, sizes and composition. It is important that the optimal EV purification method is realised in order to obtain high quality EVs to have confidence in understanding EV biology and function. In this study, the zoonotic parasite, *Fasciola hepatica*, was cultured as a case study to investigate the importance of EV purification from helminth culture media. Investigating two purification methods, it was found that size exclusion chromatography EV isolation led to a reduction of contaminating excretory-secretory and tegumental proteins. This research highlighted that purification methods do not isolate equivalent EV population profiles with similar EV purities and that size exclusion chromatography methods are likely better suited for downstream helminth EV studies and application development, compared to a differential centrifugation method.

## Introduction

Extracellular vesicle (EV) purification is challenging to standardise due to the diversity of sample composition producing EVs (cell cultures and body fluids), the need for high recovery of functional EVs, the quality of EV preparation and the simplicity of isolation [1–5]. Therefore, there is no current gold standard for EV isolation [6].

The most widely accepted method to isolate EVs involves differential centrifugation (DC). This method encompasses sequential centrifugations, increasing in speed and time, to pellet particles decreasing in size [1,2,4–11]. DC is highly reliable, yet it is a labour-intensive procedure, requiring large sample volumes to obtain low EV yields [2,4,12]. Furthermore, EV yield and purity is dependent upon DC parameters such as the rotor type, centrifugal force, centrifugation period and temperature [4,11,13]. Consequently, protocol standardisation is difficult, leading to incomparable EV characterisation and functional investigations [4,5]. To overcome EV purification challenges, many downstream EV investigations require further isolation procedures to accommodate DC, to improve sample purity and validate research analysis.

Recently, size exclusion chromatography (SEC) has been used to successfully purify EVs. SEC has been observed to separate protein aggregates and lipoproteins from EV samples as well as preclude EV or protein aggregation [1,3,4,9,14–16]. However, elution fractions containing a high yield of protein, also contain small EVs (<75 nm), meaning SEC can be selective upon EV size collection. In addition, the purest vesicle elution fraction post SEC produces low yields, so the quantity of fraction collection is dependent upon the EV purity needed for experimental investigation [14]. However, research has observed that SEC EV isolation produces greater sample purity, compared to DC and precipitating agents (Polyethylene glycol and PRotein Organic Solvent PRecipitation, PROSPR) as evidenced by EV markers (Alix and CD9) in greater concentration intensity in EV samples [2,15,17]. Potentially, SEC could be reproducible in all research settings, as it is easy to use, removes most contaminating proteins and maintains EV morphology.

Many studies in independent laboratories confirm both pathogenic flatworm and nematode helminths produce EVs, albeit during *in vitro* ex-host experimentation. Of note is the recent expansion of information on the EVs of the liver fluke *Fasciola hepatica*. Fascioliasis, infection with either *F*. *hepatica* or *F*. *gigantica*, is a major neglected zoonotic disease that infects humans and ruminant species worldwide [18,19]. At least 2.4 million people are currently infected in over seventy countries, with millions more at risk of this food borne disease [20]. Furthermore, the disease is a significant animal health and food security issue, costing the global livestock industry an excess of $3 billion *per* annum [21]. In the absence of protective vaccines, control is usually via anthelmintics, with triclabendazole (TCBZ) being the drug of choice, especially for acute disease caused by pathogenic juvenile *F*. *hepatica*. *F*. *hepatica* resistance towards TCBZ has spread widely, threatening future chemotherapeutic based control [22]. Therefore, the development of novel approaches and options for *F*. *hepatica* control must be considered a research and government priority.

The recent discovery of *F*. *hepatica* EVs identified in excretory-secretory products (ESP) has led to us re-evaluating the host-pathogen interface [23,24]. EVs of pathogen origin are enriched with pathogen molecules, thus they could potentially be utilised for improved control. In pathogens, EVs have been found to function to either promote or inhibit host immunity with recognised EV immunogenic properties highlighting EVs as vaccine preparations [25]. Furthermore, EVs have the ability to transport molecules to recipient cells, which could be utilised for drug delivery [26]. Therefore, EVs have a major role in pathogen infection and could be exploited to develop novel therapeutic approaches. Thus, it is important that the optimal EV purification method is utilised to obtain high purity EVs, which represent the whole biological EV population that would likely interact with the host environment.

Furthermore, given that all parasitic helminth EV studies completed to date have utilised DC without further EV enrichment, it is vital for the field to look at alternative EV isolation strategies [23,24,27–35]. This is particularly pertinent given the need for more functional studies into EV biology thus requiring EVs of high purity. Literature suggests that SEC is a suitable EV purification method to compare against DC purification methodology, which has been primarily used in helminthology, for the aim of investigating the most advantageous method for purifying helminth EVs from culture media for further downstream analysis, where EVs isolated are a biological representation of the helminth EV population. Therefore, in the current study, the EVs from adult *F*. *hepatica*, as a model pathogenic helminth, have been purified allowing the comparison of SEC EV purification, to the widely accepted method of DC EV preparation.

This study has discovered that SEC EV isolation leads to smaller sized and lower diversity EV populations, with importantly a higher EV purity to free ESP yield, a less diverse EV proteome and different EV gene enrichment profiles compared to DC purified EVs. Therefore, the data suggests that DC and SEC purification methods do not isolate equivalent EV population profiles and caution should be taken in the choice of EV purification utilised with functional assays incorporated into the isolation pipeline. This research has highlighted SEC methods with functional assays as the methodology of choice for helminth pathogen EV studies and application development in the absence of a gold standard purification method.

## Methods

### *F*. *hepatica* collection and culture

A local collaborating abattoir (Wales, U.K.), where animals were processed as part of the normal work of the abattoir, gave consent for the collection of live adult *F*. *hepatica* from naturally infected ovine livers of animals immediately post-slaughter, where *F*. *hepatica* were maintained as previously described [36]. Briefly, *F*. *hepatica* were thoroughly washed in phosphate buffered saline (PBS) at 37°C to remove host material. *F*. *hepatica* were then transported to the laboratory in *Fasciola* saline (Dulbecco’s modified Eagle’s medium (DMEM) (w/o NaHPO_3_ and PO_4_) plus 2.2 mM Ca (C_2_H_3_O_2_), 2.7 mM MgSO_4_, 61.1 mM glucose, 1 μM serotonin, 5 μg/mL gentamycin, 15 mM N-2-hydroxyethylpiperazine-N′-2-ethanesulfonic acid (HEPES), pH7.4) at 37°C for 1 hour. Following incubation, *F*. *hepatica* were maintained in fresh *Fasciola* saline (1 ml/ *F*. *hepatica*) at 37°C for 5 hours. All *F*. *hepatica* remained alive after *in vitro* culture incubations. Both *F*. *hepatica* and the culture media was immediately frozen and stored at -80°C, until further experimentation.

### EV purification: differential centrifugation (DC)

EVs were purified from the culture media based on the method according to Thery *et al*. [37] with slight modifications. In brief, culture media was centrifuged at 300 *x g* for 10 minutes at 4°C and then at 700 *x g* for 30 minutes at 4°C, removing any large particulates. The supernatant was ultracentrifuged at 100,000 *x g* for 80 minutes at 4°C (Optima L-100 XP ultracentrifuge (Beckman Coulter, High Wycombe, UK) using a Type 50.2 Ti rotor) and the EV depleted culture media was removed and stored at -80°C. Of note, the 10,000 *x g* centrifugation step for 30 minutes carried out by Thery *et al*. [37] was not undertaken within the current methodology to ensure isolation of the whole biological EV population. Therefore, it should be acknowledged that debris may be still present in the EV enriched sample. The pellet was washed in PBS and vortexed until the pellet had been suspended in solution. The ultracentrifugation step was repeated and the supernatant was discarded. The pellet was then re-suspended in 200 μl PBS by vortexing and stored at -80°C until further experimentation.

### EV purification: Size exclusion chromatography (SEC)

Culture media was centrifuged at 300 *x g* for 10 minutes at 4°C and then at 700 *x g* for 30 minutes at 4°C, removing any large particulates. The sample was then concentrated using 10 KDa MWCO Amicon Ultra-15 Centrifugal Filter Units (Merck Millipore), following the manufacturer’s guidelines. Briefly, the sample was centrifuged at 4000 *x g* for 20 minutes at 4°C, until approximately 500 μl of sample was retained in the filter. Filtration flow through was stored at -80°C. The sample was passed through qEVoriginal SEC columns (IZON science), utilising the manufacturer’s optimised protocol. Briefly, the column was rinsed with 10 ml of filtered (0.2 μm, Life Sciences) PBS. The sample was then added to the SEC column and the first 2.5 ml of flow through was discarded. The next 2.5 ml of flow through, containing EVs, was collected and stored at -80°C. The column was then washed with 10 ml PBS, which was combined with the filtration flow through to create EV depleted SEC ESP.

### Transmission electron microscopy (TEM)

EV samples and culture media, containing *F*. *hepatica* ESP depleted of EVs, were fixed onto formvar/carbon coated copper grids (agar scientific) by adding 10 μl sample to the grid for 45 minutes on ice. Grids were then placed on the viscous of 4% v/v uranyl acetate for five minutes on ice. Grids were stored at room temperature for at least 24 hours before being imaged using the transmission electron microscope (Jeol JEM1010 microscope at 60 kV). Images were developed and the size of 200 EVs per purification method were measured (nm) using imageJ (https://imagej.nih.gov/ij/) and statistically analysed by Mann-Whitney U test in R studio (https://www.rstudio.com/).

### Atomic force microscopy (AFM)

EV samples were diluted 1:10 with deionised water and adsorbed onto freshly cleaved mica sheets (Agar Scientific AGG250-1) for two minutes and dried under a nitrogen stream. Samples were then scanned with a Park Systems E100 AFM, using silicon probes (NT-MTD: NSG-01 & NSG-03PT) in non-contact mode. Topographic height and phase images were scanned at 512 x 512 pixels at a rate of 0.2 Hz. Images were analysed using Gwyddion software (www.gwyddion.net/).

### Sodium dodecyl sulfate polyacrylamide gel (SDS-PAGE) electrophoresis

*F*. *hepatica* somatic sample was prepared by homogenising *F*. *hepatica* in homogenisation buffer (20 mM potassium phosphate buffer (pH 7.4), 0.1% (v/v) triton X-100 and protease inhibitor tablets, EDTA-free) (0.5 ml/ *F*. *hepatica*) on ice, before centrifuging at 21,000 *x g* at 4°C for 30 minutes. The supernatant was termed the cytosolic fraction, and was stored at -20°C until further experimentation.

Culture media, containing *F*. *hepatica* ESP depleted from EVs from both purification methods, was precipitated with an equal volume of ice-cold 20% (w/v) trichloroacetic acid (TCA) in acetone for one hour at -20°C. The sample was centrifuged at 21,000 x *g*, 4°C for 20 minutes. The pellet was washed twice in ice-cold acetone with centrifugation 21,000 x *g*, 4°C for 20 minutes between washes. The pellet was left to air dry at -20°C for 15 minutes, before being resuspended in PBS and stored at -20°C until further experimentation.

EV samples were centrifuged at 100,000 x *g* at 4°C for 30 minutes and the supernatant was discarded. Lysis buffer (PBS, 0.1% (v/v) triton X-100 and protease inhibitor tablets, EDTA-free) was added to the pellet and the sample was sonicated for 30 seconds and then rested on ice for 30 seconds and repeated three times. Following lysis, the sample was centrifuged at 100,000 x *g* at 4°C for 30 minutes and the soluble fraction in the supernatant was TCA precipitated as above and stored at -20°C until further use. The insoluble fraction was washed following the protocol of Hart *et al*. [38]. Briefly, the pellet was re-suspended in sodium carbonate buffer (0.1 M Na_2_CO_3_ (pH 11), 10 mM EDTA, 20 mM DTT and protease inhibitor tablets, EDTA-free), vortexed and left for one hour at 4°C. The sample was then centrifuged at 100,000 x *g* at 4°C for 30 minutes and the supernatant was discarded. The pellet was washed in sodium carbonate buffer, vortexed, left for 30 minutes at 4°C and centrifuged as previously described. This wash step was repeated before the pellet was re-suspended in solubilisation buffer (20 mM potassium phosphate (pH 7.4), 4% SDS (w/v) and protease inhibitor tablets, EDTA-free). The sample was heated to 95°C for five minutes and then stored at -20°C until further experimentation.

Samples were quantified using either the bicinchoninic acid assay (Thermo scientific) or Bradford assay (Sigma), following the manufacturer’s protocol. Comparisons between sample protein concentrations were statistically analysed using Kruskal-Wallis test with the Dunn’s Post-hoc test using Sidak correction in R package (https://www.r-project.org/), where significance was considered p<0.05. Laemmli protein 4 x sample buffer (Bio-rad) was added to the sample (3:1 ratio) and heated to 95°C for 10 minutes. The samples were then loaded into 7 cm 12.5% Tris/glycine polyacrylamide gels and run using the Protean III system (Bio-Rad). Gels were run at 70 V, until the dye front passed through the stacking gel, and then the voltage was increased to 150 V until completion. Gels were fixed (40% ethanol (v/v), 10% acetic acid (v/v)) and stained with colloidal Coomassie Brilliant blue (Sigma). Gels were imaged using the GS-800 calibrated densitometer (Bio-rad).

### Western blotting

Samples were prepared and run upon SDS-PAGE gels as previously mentioned. Gels were electrophoretically transferred onto Hybond-C extra nitrocellulose paper (GE Healthcare) using a Trans-Blot Cell at 40 V for two hours in transfer buffer (192 mM Glycine, 25 mM Tris-HCL (pH 8.3), 20% (v/v) methanol) according to the method of Towbin *et al*. [39]. Transfer efficiency was determined by staining the membrane with amido black (0.1% amido black (w/v), 10% acetic acid (v/v), 25% isopropanol (v/v)) for one minute. The membrane was then destained (10% acetic acid (v/v) and 25% isopropanol (v/v)), and washed three times in Tris buffered saline (100 mM Tris-HCL (pH 7.5), 0.9% sodium chloride (w/v)) with 1% Tween 20 (v/v) (TTBS). The membrane was then blocked overnight in TTBS and 5% skimmed milk powder (v/v) on a rocker at 4°C.

Primary antibody, either anti-glutathione transferase sigma class (Anti-FhGST-S1) at 1:20,000 [40], Anti-*Fasciola* cathepsin L1 at 1:6,000 (Anti-FhCat-L1) which was commercially made (Lampire) from polyclonal antibodies to a purified recombinant cathepsin L1 from *F*. *hepatica* expressed in yeast and raised in rabbits or Anti-fatty acid binding protein V (Anti-FhFABP-V) at 1:2,000 which was commercially made (Lampire) from polyclonal antibodies to a purified recombinant fatty acid binding protein V from *F*. *hepatica* expressed in yeast and raised in rabbits, in TTBS and 1% skimmed milk (v/v) was added to blocked membranes. Anti-FhGST-S1 and Anti-FhCat-L1 primary antibodies are known to have high specificity however, Anti-FhFABP-V is known to be reactive to FABP I, FABP II, FABP III and FABP V. The membrane was rocked for one hour at room temperature. The membrane was then washed three times for five minutes in TBS. The secondary antibody (IgG, anti-rabbit IgG (whole molecule) conjugated to alkaline phosphatase (AKP, Sigma) dilution 1: 30,000) in TTBS was then added to the membrane and rocked for one hour at room temperature. Membrane washing was then repeated as previously described. Recognised proteins were visualised using 5-bromo-4-chloro-3-indoyl phosphate/nitro blue tetrazolium liquid substrate system. In brief, 33 μl 5-bromo-4-chloro-3-indoyl phosphate (50 mg/ml) and 330 μl nitro blue tetrazolium (10 mg/ml) was added to 10 ml substrate buffer (0.1 M Tris, 100 mM sodium chloride, 5 mM magnesium chloride, (pH 9.5)). The detection solution was added to the membrane on the rocker at room temperature until either visualisation of banding occurred or up to five minutes. The reaction was stopped by water washes. Membranes were scanned using the GS-800 calibrated densitometer (Bio-rad).

### In-gel tryptic digestion, mass spectrometry and protein identification

All protein bands were excised from one dimensional SDS-PAGE electrophoresis and digested as described in Morphew *et al*. [41]. Briefly, protein bands were washed in 50% (v/v) acetonitrile and 50% (v/v) 50 mM ammonium bicarbonate at 37°C until destained. Destained bands were dehydrated in 100% acetonitrile at 37°C for 15 minutes, before being removed and dried at 50°C. Protein bands were then incubated with 10 mM dithiothreitol in 50 mM ammonium bicarbonate for 30 minutes at 80°C. The supernatant was discarded before bands were incubated with 55 mM iodoacetamide in 50 mM ammonium bicarbonate for 20 minutes at room temperature in the dark. The supernatant was discarded and the bands were washed twice for 15 minutes at room temperature with 50% (v/v) acetonitrile and 50% (v/v) 50 mM ammonium bicarbonate. Excess was removed before 100% acetonitrile was added to the bands at room temperature for 15 minutes. The supernatant was removed and bands were dried at 50°C. Bands were rehydrated and digested using 50 mM ammonium bicarbonate containing trypsin (modified trypsin sequencing grade, Roche, UK) at 10 ng/μl at 37°C overnight. The supernatant was stored, before 100% acetonitrile was added to the bands at room temperature for 15 minutes, followed by adding 50 mM ammonium bicarbonate to the bands at room temperature for 15 minutes. This step was repeated and from each step, the supernatant was removed and pooled for each band. The 100% acetonitrile step was repeated, and supernatant pooled, before the samples were vacuum dried (Maxi dry plus, Heto) and re-suspended in 20 μl of 0.1% (v/v) formic acid for tandem mass spectrometry.

Liquid Chromatography tandem mass spectrometry (Agilent 6550 iFunnel Q-TOF) coupled to a HPLC-Chip (1200 series, Agilent Technologies, Cheshire, UK) was used for peptide separations. The HPLC-Chip/Q-TOF system was equipped with a capillary loading pump (1200 series, Agilent Technologies) and a nano pump (1200 series, Agilent Technologies). Sample injection was conducted with a micro auto sampler (1100 series, Agilent Technologies), where 1 μl of sample in 0.1% formic acid was loaded on to the enrichment column at a flow of 2.5 μL/min followed by separation at a flow of 300 nL/min. A Polaris Chip was used (G4240-62030, Agilent Technologies), comprising a C18 enrichment/trap column (360 nl) and a C18 separation column (150 mm x 75 Âμm), where ions were generated at a capillary voltage of 1950 V. The solvent system was: solvent A (ultra-pure water with 0.1% formic acid), and solvent B (90% acetonitrile with 0.1% formic acid). The liquid chromatography was performed with a piece-linear gradient using 3–8% of solvent B over 0.1 minutes, 8–35% solvent B over 14.9 minutes, 35–90% solvent B over five minutes and hold at 90% solvent B for two minutes. Tandem mass spectrometry was performed in AutoMS2 mode in the 300–1700 Da range, at a rate of 5 spectra per second, performing MS2 on the 5 most intense ions in the precursor scan. Masses were excluded for 0.1 minutes after MS/MS was performed. Reference mass locking was used for internal calibration using the mass of 391.2843 Da.

Peak lists were generated with Mass Hunter Qualitative Analysis software (V B.06, Agilent Technologies) and exported as Mascot Generic Files. Samples were processed following Morphew *et al*. [42]. Briefly, samples were submitted to MASCOT daemon (Matrix Science, v2.4.1) MS/MS ions search against *F*. *hepatica* gene sequences, accessed through WormBase ParaSite (http://parasite.wormbase.org/, accession PRJEB6687, version WBPS9). Search parameters included setting the enzyme to trypsin with one missed cleavage allowed, setting fixed modifications to carbamidomethylation with variable modifications set for oxidation of methionine, fixing monoisotopic masses with unrestricted protein masses with peptide and fragment mass tolerances at ±1.2 Da and ±0.6 Da respectively (project accession PXD008737). Protein identifications were reported at a false discovery rate of 1%. For the overall list of proteins identified (data in S1 File), only proteins with at least 2 unique peptides and present in both biological replicates (n = 2) were selected. Protein sequences were searched using BLAST2GO (https://www.blast2go.com/) obtaining BLAST descriptions and gene ontology terms (data in S2 File). Gene ontology enrichment analysis was completed using GOATOOLS python package (https://github.com/tanghaibao/goatools) (data in S4 File) where the GO terms were not propagated up the hierarchy and p<0.05 identified significance.

## Results

### EV morphological characteristics

**Fig 1:**
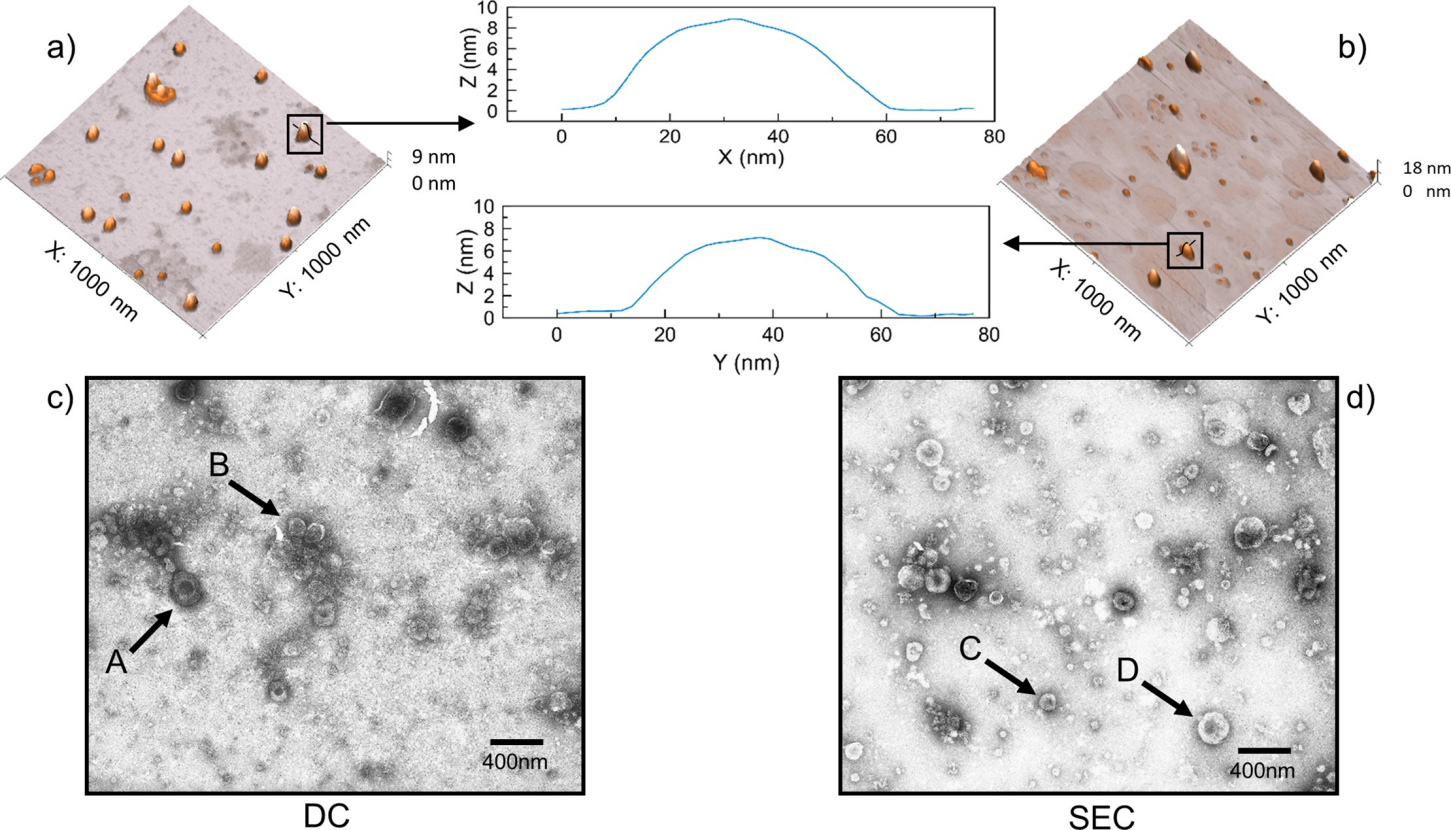
Topography profiles and micrographs of extracellular vesicles purified via differential centrifugation and size exclusion chromatography. Representative atomic force microscopy topography profiles (A and B) and transmission electron microscopy images (C and D) of extracellular vesicles purified from adult *F*. *hepatica* via differential centrifugation (A and C) and size exclusion chromatography (B and D). The central graphs represent individual extracellular vesicle topography from each purification method as determined by atomic force microscopy. Profiles identified that extracellular vesicle structures were similar using both differential centrifugation and size exclusion chromatography purification methods although size exclusion chromatography purified extracellular vesicles were significantly smaller (76 nm ± 44 SD) than differential centrifugation extracellular vesicles (95 nm ± 58 SD) (W = 14,726, p < 0.001) and differential centrifugation purified extracellular vesicles with a greater range of extracellular vesicle sizes than size exclusion chromatography (differential centrifugation range = 505 nm, size exclusion chromatography range = 285 nm). Arrows on TEM images highlight different sized EVs where A = 130.5 nm, B = 235.0 nm, C = 111.7 nm and D = 226.4 nm.

In order to assess EV morphology post DC and SEC purification, both atomic force microscopy (AFM) and transmission electron microscopy (TEM) imaging were utilised. AFM and TEM identified that EV structures were characteristically diverse in size and morphology using both DC and SEC purification methods, representing a biological population of EVs (Fig 1). When comparing EV dimensions following TEM analysis, SEC EVs were significantly smaller (76 nm ± 44 SD) than DC EVs (95 nm ± 58 SD) (n = 200) (W = 14,726, p < 0.001) and DC purified EVs displayed a greater range of EV sizes than SEC (DC range = 505 nm, SEC range = 285 nm). All observed EVs were intact in TEM images for both purification methods. Aggregation of EVs and contaminants in the image background was observed using both purification methods in AFM and TEM images.

TEM micrographs produced for the culture media containing *F*. *hepatica* ESP depleted of EVs identified few EVs (data not shown). In addition, EVs were not found within the ultrafiltration flow through during the SEC purification method (data not shown). In all non-EV preparations debris and additional non-EV contaminants were identified.

### EV comparative protein concentrations

**Fig 2:**
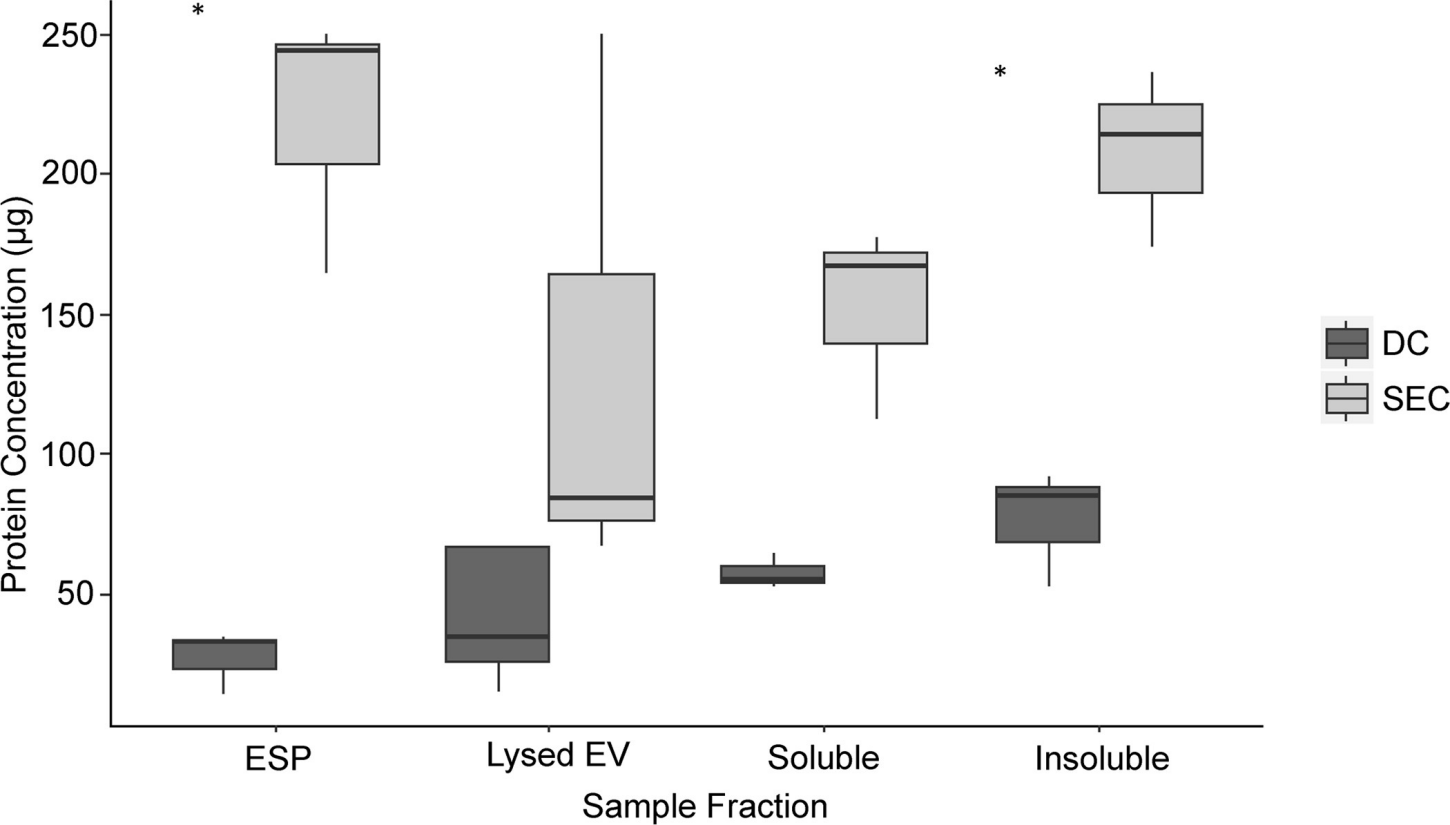
Protein quantification of extracellular vesicle preparation fractions from differential centrifugation and size exclusion chromatography purification. Fractions produced from either differential centrifugation or size exclusion chromatography methods (excretory-secretory protein, whole lysed extracellular vesicle, soluble extracellular vesicle protein and insoluble extracellular vesicle protein) were assayed for protein levels and statistically analysed (Kruskal-Wallis test, with Dunn’s Post-hoc test using Sidak correction) for differences. Asterisks identify significance where p < 0.05.

EV preparations obtained from both DC and SEC purification methods were quantified for protein content for ESP (protein content for residual ESP after EV purification), whole lysed EV samples and soluble and insoluble EV fractions (Fig 2). All samples quantified were unrelated biological replicates. Analysis of the EV preparation via the DC purification method revealed comparable protein quantities between ESP (average = 30 μg, n = 3) and whole lysed EV samples (average = 40 μg, n = 3), even though the whole lysed EV samples had greater quantity variation (ESP range = 20 μg, whole lysed EV range = 140 μg). However, this protein profile was not replicated for SEC derived EV preparations. Protein quantity of SEC ESP was nearly threefold that of the lysed EV samples (ESP average = 240 μg, n = 3, whole lysed EV average = 80 μg, n = 3), even though whole lysed EV samples had a greater quantity variation (ESP range = 90 μg, whole lysed EV range = 180 μg). Furthermore, SEC purified samples produced a higher protein yield than DC in both ESP and whole lysed EV samples, with ESP showing a significant increase (D = -3.13, p = 0.02, n = 3). Similarly, SEC produced a greater protein yield of soluble and insoluble EV fraction protein (soluble protein average = 170 μg, n = 9, insoluble protein average = 210 μg, n = 9) than DC (soluble protein average = 30 μg, n = 9, insoluble protein average = 60 μg, n = 9). Both EV purification methods showed that there was a greater protein quantity of insoluble EV protein compared to soluble EV protein, with the SEC soluble fraction containing significantly less protein than insoluble protein fraction (D = -0.43, p = 0, n = 9).

### EV comparative proteome analysis

**Fig 3:**
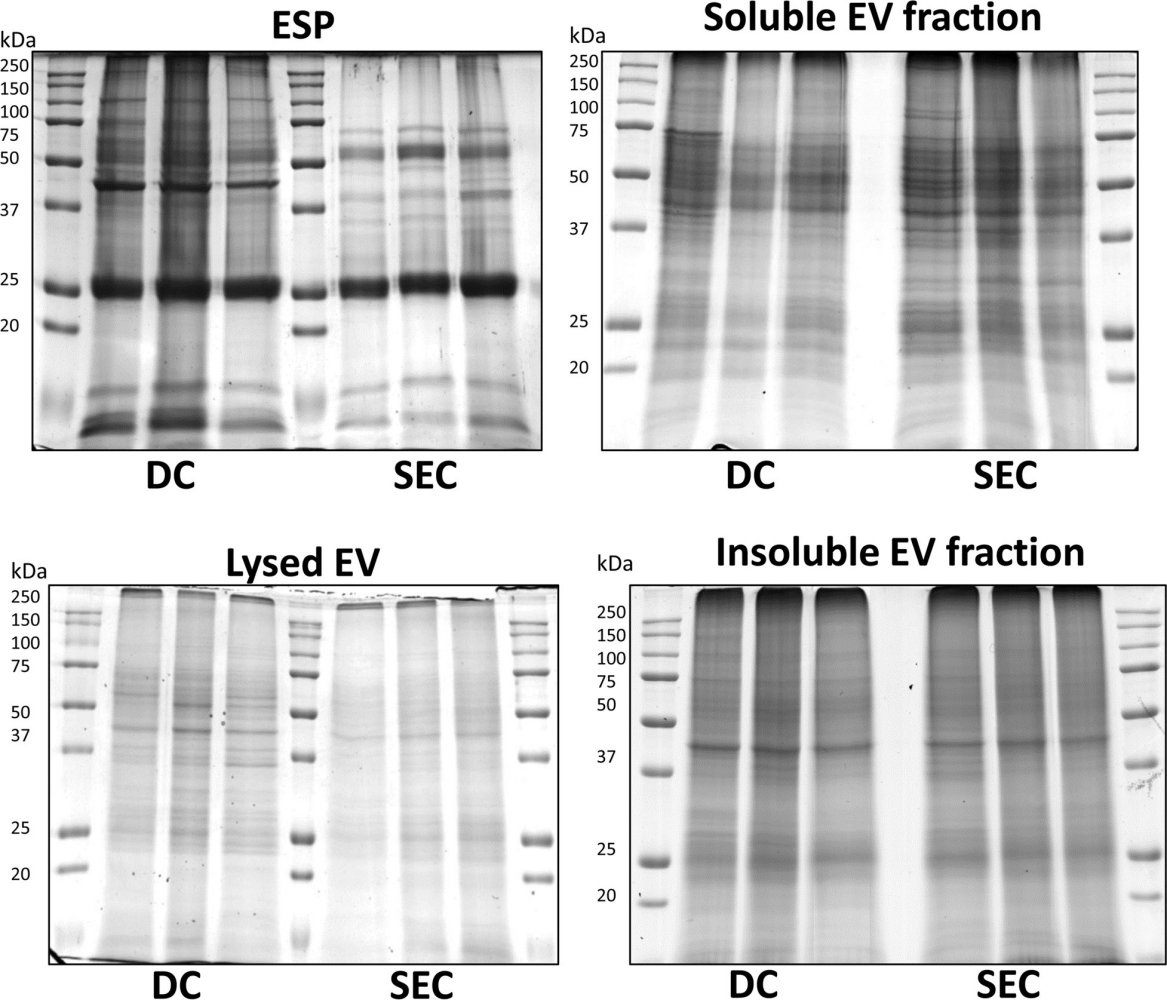
Protein profiles of extracellular vesicle fractions from differential centrifugation and size exclusion chromatography purification. Biological triplicate protein profiles as observed by one dimensional SDS-PAGE electrophoresis of excretory-secretory protein (10 μg protein), whole lysed extracellular vesicle (10 μg protein), soluble extracellular vesicle protein (20 μg protein) and insoluble extracellular vesicle protein (20 μg protein). All proteins were run on 12.5% SDS-PAGE electrophoresis gels and Coomassie blue stained.

Whole lysed EV and ESP samples displayed different protein profiles as observed by one dimensional sodium dodecyl sulfate-polyacrylamide gel (SDS-PAGE) electrophoresis, suggesting that both EV purification methods selectively isolate EVs away from *F*. *hepatica* secretions or ESP (Fig 3). In addition, biological replication of EV preparations indicated that protein quantification and preparation was reproducible as all sample replicates produced comparable protein banding patterns. Comparing lysed EV preparation protein profiles using DC and SEC purification methods, banding differences were identifiable, especially between 50–100 kDa and 25–37 kDa markers. Differences were less noticeable within the protein profiles of the soluble and insoluble EV fraction proteins and between the method by which the EVs were purified.

Following SDS-PAGE, mass spectrometry was employed to identify key differences between the profiles from a combined analysis of the soluble and insoluble EV protein fractions from SEC and DC isolated EVs. Following analysis, SEC purification revealed 68% protein matching between the soluble and insoluble SEC EV fractions with a 63% similarity of proteins for DC purified soluble and insoluble fractions. Following the combination of the soluble and insoluble protein identifications the whole SEC and DC purified EV proteomes were further analysed to identify key protein differences between the purification methods. Interestingly, 77% of proteins were comparable between SEC biological replicates and 77% between DC biological replicates.

In DC purified EVs, 392 proteins were identified, while 321 proteins were observed using SEC purification across both replicates. Of these proteins, 276 were comparable between purification methods demonstrating differences and similarities between the two methodologies. When looking at unique proteins to each method, 116 proteins were found exclusively in DC purified EVs and 45 proteins were found exclusively in SEC purified EVs (data in S5 File). Gene ontology analysis identified that EV proteins mostly showed biologically function in organic substance and primary metabolic processes as well as single-organism cellular processes irrespective of EV purification method (S6 File). These categories encompass proteins such as cathepsin L and GAPDH. However, there were significant differences in gene ontology terms such as catabolic process and microtubule-based process which were only found in DC purified EVs, while many more ‘regulatory-cell invasion’ terms (i.e. cellular localisation and response to stress) were only found in SEC purified EVs. Catabolic process and microtubule-based process only found in DC purified EVs refers to proteins such as Mov34/MPN/PAD-1 family protein, ubiquitin—protein ligase and alpha-tubulin. While, ‘regulatory-cell invasion’ terms only found in SEC purified EVs includes ADP-ribosylation factor family protein, T-complex protein 1 zeta subunit and glutathione peroxidase. Considering molecular function, transmembrane transporter activity and substrate-specific transporter activity gene ontology terms were only identified in SEC purified EVs including proteins such as IC domain protein HAD ATPase P-type family and hypothetical proteins. For cellular components, catalytic complex and intrinsic component of the membrane were the only categories found uniquely in EVs purified from DC including multicatalytic endopeptidase, ATPase family protein and proteasome subunit alpha domain protein and cell periphery, cell projection, plasma membrane, plasma membrane part and plasma membrane region were the only unique components found in EVs purified from SEC including IC domain protein, HAD ATPase, P-type family, ADP-ribosylation factor family protein and hypothetical proteins.

When specifically looking at protein identifications, DC EVs consistently demonstrated a greater amount of unique peptides hit per protein than SEC. The top 50 proteins found in EVs purified using SEC were also present in the DC EV purified proteome. However, proteins relating to gene scaffolds BN1106_s90B000599 (ATPase family protein), BN1106_s1277B000102 (HSP90 protein), BN1106_s63B000395 (hypothetical protein D915_01544) and BN1106_s285B000846 (unnamed protein product) were found in the top 50 proteins of DC purified EVs, but were not present in the SEC isolated EV proteome (highlighted within Table 1). Common EV markers from ExoCarta database (http://www.exocarta.org/) present in the top 50 EV proteins using both purification methods included heat shock proteins, glyceraldehyde-3-phosphate dehydrogenase, actin, 14-3-3 protein, annexin and tubulin. Unique EV markers, gelosin and phosphoglycerate kinase were found in the top 50 EV protein hits using DC purification, while a tetraspanin EV marker was found in the top 50 protein hits using SEC purified EVs.

**Table 1:** Top 50 extracellular vesicle protein accessions purified by differential centrifugation and size exclusion chromatography. BLAST descriptions were chosen upon the lowest E-value hit and top accessions were identified by the highest unique peptide hits. Data analysed using BLAST2GO. Highlighted cells in grey shading signify proteins identified in the top 50 proteins of DC or SEC purified EVs, but were not present in the full EV proteome of the opposite purification methodology. Full protein identifications can be found in S3 File.

Size Exclusion Chromatography					Differential Centrifugation				
Rank	Sequence ID	Description	Unique Peptide Hits	Total Peptide Hits	Rank	Sequence ID	Description	Unique Peptide Hits	Total Peptide Hits
1	BN1106_s3182B000117	myosin heavy chain	115	437	1	BN1106_s3182B000117	myosin heavy chain	135	605
2	BN1106_s309B000234	heat shock protein 70	109	1659	2	BN1106_s309B000234	heat shock protein 70	120	1906
3	BN1106_s1300B000145	SJCHGC06288 protein	100	1994	3	BN1106_s1300B000145	SJCHGC06288 protein	107	2046
4	BN1106_s175B000200	hexokinase	97	1440	4	BN1106_s1320B000236	heat shock protein 90	104	843
5	BN1106_s819B000364	annexin A7	96	1480	5	BN1106_s819B000364	annexin A7	99	1812
6	BN1106_s3585B000136	myoferlin	95	669	6	BN1106_s4069B000247	alpha-actinin	98	880
7	BN1106_s617B000566	leucine amino peptidase	94	1528	7	BN1106_s617B000566	leucine amino peptidase	97	1460
8	BN1106_s4069B000247	alpha-actinin	87	633	8	BN1106_s1403B000129	hypothetical protein	94	1627
9	BN1106_s2907B000133	hypothetical protein	86	1441	9	BN1106_s1871B000313	programmed cell death 6-interacting protein	93	902
10	BN1106_s1403B000129	hypothetical protein	82	964	10	BN1106_s2907B000133	hypothetical protein	92	1777
11	BN1106_s274B000296	Adenosine Triphosphate binding cassette subfamily	82	749	11	BN1106_s175B000200	hexokinase	92	1344
12	BN1106_s945B000218	Annexin	81	663	12	BN1106_s3585B000136	myoferlin	90	736
13	BN1106_s3227B000227	phosphopyruvate hydratase	78	567	13	BN1106_s90B000599	Adenosine Triphosphase family protein	89	200
14	BN1106_s2471B000098	PREDICTED: phosphatidylcholine translocator ABCB4	77	635	14	BN1106_s2471B000098	PREDICTED: phosphatidylcholine translocator ABCB4	86	572
15	BN1106_s1320B000236	heat shock protein 90	77	521	15	BN1106_s3904B000042	14-3-3 protein	83	1029
16	BN1106_s1871B000313	programmed cell death 6-interacting protein	76	851	16	BN1106_s606B000246	alpha-tubulin	83	787
17	BN1106_s233B000262	hypothetical protein	74	527	17	BN1106_s3227B000227	phosphopyruvate hydratase	82	598
18	BN1106_s246B000252	phosphoenolpyruvate carboxykinase	69	602	18	BN1106_s246B000252	phosphoenolpyruvate carboxykinase	81	909
19	BN1106_s204B000249	calpain	69	546	19	BN1106_s945B000218	Annexin	80	712
20	BN1106_s2349B000188	severin	69	462	20	BN1106_s7079B000034	hypothetical protein	80	615
21	BN1106_s3261B000048	otoferlin	65	360	21	BN1106_s2697B000090	leucine Rich repeat-containing domain protein	80	500
22	BN1106_s1081B000242	chain A, Sigma class glutathione S-transferase	64	472	22	BN1106_s274B000296	Adenosine Triphosphate binding cassette subfamily	78	669
23	BN1106_s500B000161	annexin	63	413	23	BN1106_s233B000262	hypothetical protein	76	484
24	BN1106_s3904B000042	14-3-3 protein	62	846	24	BN1106_s2907B000132	actin	75	805
25	BN1106_s2615B000090	dysferlin	61	449	25	BN1106_s1153B000359	tubulin beta-4	71	619
26	BN1106_s468B000343	xaa-pro dipeptidase	60	851	26	BN1106_s2349B000188	severin	70	1062
27	BN1106_s3747B000112	hypothetical protein	60	529	27	BN1106_s4469B000065	fructose-bisphosphate aldolase class I	68	622
28	BN1106_s4252B000085	uncharacterized protein	60	475	28	BN1106_s5174B000030	glyceraldehyde-3-phosphate dehydrogenase	68	408
29	BN1106_s658B000223	hypothetical protein	58	825	29	BN1106_s3261B000048	otoferlin	68	386
30	BN1106_s7079B000034	hypothetical protein	58	531	30	BN1106_s658B000223	hypothetical protein	67	929
31	BN1106_s686B000273	14-3-3 protein	54	480	31	BN1106_s500B000161	Annexin	67	544
32	BN1106_s3266B000046	annexin	54	381	32	BN1106_s4860B000047	Tubulin beta chain	66	748
33	BN1106_s2091B000373	DnaK family protein	54	278	33	BN1106_s2615B000090	Dysferlin	66	534
34	BN1106_s1515B000336	filamin-C	52	139	34	BN1106_s1277B000102	Heat shock protein 90	65	148
35	BN1106_s5174B000030	glyceraldehyde-3-phosphate dehydrogenase	51	243	35	BN1106_s468B000343	Xaa-Pro dipeptidase	64	766
36	BN1106_s2907B000132	actin	50	441	36	BN1106_s204B000249	calpain	64	431
37	BN1106_s3172B000053	14-3-3 protein	49	513	37	BN1106_s4413B000122	hypothetical protein	64	421
38	BN1106_s4469B000065	fructose-bisphosphate aldolase class I	49	431	38	BN1106_s63B000395	hypothetical protein	63	184
39	BN1106_s617B000567	leucyl aminopeptidase	48	507	39	BN1106_s3172B000053	14-3-3 protein	62	545
40	BN1106_s2100B000128	hypothetical protein	48	301	40	BN1106_s4252B000085	uncharacterized protein	61	524
41	BN1106_s1191B000313	hypothetical protein	48	183	41	BN1106_s3747B000112	hypothetical protein	60	584
42	BN1106_s3001B000132.	hypothetical protein	47	255	42	BN1106_s1081B000242	chain A, Sigma class glutathione S-transferase	59	369
43	BN1106_s6821B000024	hypothetical protein	45	351	43	BN1106_s617B000567	leucyl aminopeptidase	58	457
44	BN1106_s55B000373	hypothetical protein	44	97	44	BN1106_s686B000273	14-3-3 protein	57	468
45	BN1106_s584B000346	glucose transporter-2 protein	43	260	45	BN1106_s2091B000373	DnaK family protein	57	283
46	BN1106_s4860B000047	tubulin beta chain	42	235	46	BN1106_s3033B000087	phosphoglycerate kinase	57	231
47	BN1106_s584B000350	glucose transporter-2 protein	41	320	47	BN1106_s285B000846	unnamed protein product	56	442
48	BN1106_s4560B000072	tetraspanin family protein	41	223	48	BN1106_s6821B000024	hypothetical protein	55	437
49	BN1106_s1614B000280	alkyl hydroperoxide reductase/ Thiol specific antioxidant family	41	204	49	BN1106_s2349B000191	gelsolin repeat protein	54	286
50	BN1106_s487B000135	hypothetical protein	41	180	50	BN1106_s487B000135	hypothetical protein	52	177

EV proteins unique to SEC or DC purification were further investigated to assess the likelihood of non-EV contamination in each preparation likely from *F*. *hepatica* ESP or the tegument using data from recent proteomic studies (data in S5 File) [23,24,43–46]. Of the 45 proteins identified in SEC purified EVs only, 7 proteins were observed previously in ESP with 11 proteins identified previously in tegumental preparations. Looking at the 116 proteins identified in DC purified EVs only, 11 proteins were previously observed in ESP proteomic studies with an additional 27 proteins located in tegumental proteomic studies. Of note is the reduced abundance of the potential tegumental proteins in the SEC preparation in comparison to DC EV preparations. Based on the number of unique or total peptides for each protein as an relative assessment of abundance, potential tegument identifications from DC prepared EVs were in greater quantities (Average of 14 unique peptides and 47 total peptides per protein) compared to EVs from SEC preparations (Average of 9 unique peptides and 15 total peptides per protein).

Of interest in the DC prepared EVs was the identification of 5 proteins that have previously been well documented in the proteome of *F*. *hepatica* eggs. Specifically, two ferritin-like proteins (BN1106_s709B000642 & BN1106_s709B000627), heat shock protein-35a (BN1106_s7572B000046), oxidoreductase (BN1106_s3715B000086) and an omega class glutathione S-transferase (BN1106_s1029B000154) which were all identified within DC EVs only.

### Comparative gene enrichment

Gene enrichment was undertaken to determine significant over-representation of biological characteristics within the DC and SEC purified proteomes, compared to the *F*. *hepatica* genome set. Gene enrichment analysis revealed enriched gene ontology terms for both purification methods in biological process, molecular function and cellular component categories against the *F*. *hepatica* genome (S7 File). Several gene ontology terms were enriched, which were not represented in EVs from both purification methods. For example, in biological processes, ATP synthesis coupled proton transport, carbohydrate metabolic process, cellular iron ion homeostasis, DNA replication, gluconeogenesis, iron ion transport, protein transport, proton transport, regulation of protein phosphorylation, DNA templated transcription and transmembrane transport were enriched in DC purified EVs only, where categories included proteins such as ATP synthase F1 beta subunit and peptidase T1 family. While nucleocytoplasmic transport, nucleosome assembly, oxidation-reduction process, phosphatidylinositol metabolic process, protein dephosphorylation, proteolysis, regulation of actin filament polymerization, small GTPase mediated signal transduction and translation including proteins such as Ras family protein and aldehyde dehydrogenase family protein were not enriched in DC purified EVs. Pairwise comparisons of DC purified EVs compared to SEC purified EVs demonstrated that, when considering biological processes, there were significantly less translation gene ontology terms (p = 0.02) and significantly more DNA-templated regulation of transcription gene ontology terms (p = 0.04), when considering cellular components there were significantly less ribosome gene ontology terms (p = 0.04) and when considering molecular function, structural constituents of the ribosome gene ontology terms (p = 0.02) were significantly less enriched.

### Comparative Western blotting

**Fig 4:**
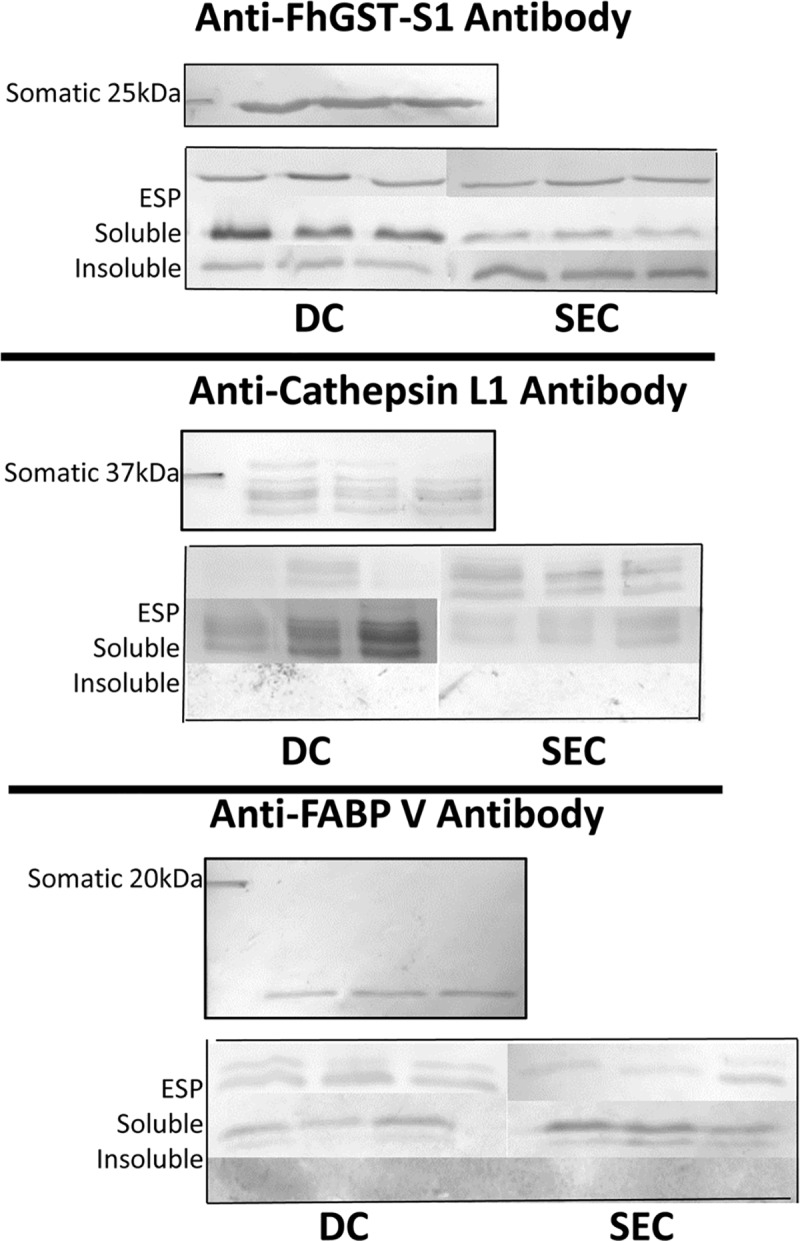
Extracellular vesicle protein preparation Western blot profiles purified using differential centrifugation and size exclusion chromatography. Extracellular vesicle preparation western blot profiles of somatic (10 μg protein), excretory-secretory protein (10 μg protein), soluble extracellular vesicle protein (20 μg protein) and insoluble extracellular vesicle protein (20 μg protein). Biological triplicates were analysed on 12.5% SDS-PAGE electrophoresis gels and transferred to nitrocellulose membranes for immune recognition. Samples of extracellular vesicle preparations purified using either the differential centrifugation method or size exclusion chromatography method were probed with anti-glutathione transferase sigma class (Anti-FhGST-S1), Anti-*Fasciola* cathepsin L1 (Anti-FhCat-L1) or anti-fatty acid binding protein V (Anti-FhFABP-V).

The relative abundances of three proteins, which are well recognised to be located in *F*. *hepatica* ESP, EVs and somatic fractions, were compared utilising Western blotting. Somatic, ESP, soluble and insoluble EV fraction samples were assessed using both DC and SEC EV purification approaches (Fig 4). Equal concentrations of each protein sample was assayed for each EV purification Western blot. Anti-fatty acid binding protein V (Anti-FhFABP-V) antibody recognition was observed within somatic, ESP and soluble EV fractions using both purification methods but not within the insoluble EV fraction yet, DC EV purification demonstrated a marginally higher anti-FABP V recognition in EV soluble protein concentrations than SEC methods. Recognition with anti-glutathione transferase sigma class (Anti-FhGST-S1), identified in all fractions (somatic, ESP, insoluble and soluble EV), was notably observed to a greater extent within the insoluble EV fraction using SEC purification rather than DC purification.

Anti-*Fasciola* cathepsin L1 (Anti-FhCat-L1) antibody recognition was identified in all fractions (somatic, ESP and soluble EV) with the exception of insoluble EV preparations. Of note is the increased recognition of *Fasciola* cathepsin L1 observed in the soluble EV fraction of DC purified EVs when compared to SEC EVs. Furthermore, there was greater recognition by Anti-FhCat-L1 in the ESP following SEC purification rather than DC. To further investigate the abundance of cathepsin L proteases in DC and SEC EVs the proteomic data sets generated in the current work were examined to reveal which cathepsin L proteases were identified in the respective EV preparations (Table 2). Cathepsin L protease identifications revealed a greater number of CL1 clade (CL1A and CL1D) identified in EVs purified by SEC, rather than EVs purified by DC. In addition, DC purified EVs contained a greater number of the CL5 clade members.

**Table 2:** Cathepsin L proteases identified in extracellular vesicles purified by differential centrifugation and size exclusion chromatography. Protein accession numbers were chosen upon the lowest E-value hit and top accessions were identified by the highest unique peptide hits. Protein clades were identified from references [24,41,47,48].

			Size Exclusion Chromatography		Differential Centrifugation	
Sequence ID	Protein Accession	Description [species]	Unique Peptide Hits	Total Peptide Hits	Unique Peptide Hits	Total Peptide Hits
BN1106_s8490B000026	AAB41670.2	secreted cathepsin L1A [*Fasciola hepatica*]	29	109	23	91
BN1106_s4636B000039	AAF44676.1	cathepsin L5 [*Fasciola gigantica*]	-	-	9	13
BN1106_s10332B000011	ACJ12893.1	cathepsin L1D [*Fasciola hepatica*]	14	43	8	22
BN1106_s7289B000014	AAB41670.2	secreted cathepsin L1A [*Fasciola hepatica*]	12	19	-	-
BN1106_s8098B000020	ASK40163	ProCathepsin L2 [*Fasciola hepatica*]	10	21	9	22
BN1106_s7456B000012	ABV90502.1	cathepsin L1D, partial [*Fasciola gigantica*]	6	12	-	-
BN1106_s6354B000017	ATW63990.1	Pro cathepsin L5 [*Fasciola hepatica*]	5	10	6	14
BN1106_s10332B000010	ACJ12894.1	cathepsin L1D [*Fasciola hepatica*]	5	8	-	-

## Discussion

Given recent discoveries that EVs function in host-pathogen communication promoting or inhibiting host immunity, it is vital that reliable protocols for EV isolation, away from ESP, are conducted for downstream studies and application development. Therefore, the current work aimed to assess the current standard procedure for helminth pathogen EV analysis, DC, with a SEC approach well-established outside of the pathogen field when purifying EVs from culture media.

EV morphological characteristics assessed after both DC and SEC purification methods, identified that SEC purified EVs on average were smaller (76 nm ± 44 SD) and less diverse in size than DC purified EVs (95 nm ± 58 SD), indicating different EV populations were likely isolated by each method. DC purified EV size and diversity (27 nm and above) were similar to EV size predictions made by the centrifugation calculator [49]. Centrifugation calculators further predict that 38% of free protein will be purified in solution using DC purification methods. Furthermore, DC of EVs is more successful when sedimentation coefficients of the particles to be distinguished differ significantly [49]. For example, different centrifugation forces are required to obtain optimal EV to protein yield for different cell lines [50]. Thus, the difficulty of DC based standardisation across laboratory settings and sample types is becoming clearer.

During the current analysis, EVs were observed to aggregate together and contaminants co-purified were visible utilising both purification methods. However, Nordin *et al*. [2] demonstrated that DC purified EVs were fused, disrupted (62%) and aggregated when compared to SEC purified EVs yet, in the current investigation, EVs from either purification method were not damaged. It has been noted that forced filtration of samples can cause EV rupture and deformity, so sample preparation and SEC methods are advised to be performed using gravity only methods [1,6,11]. Aggregates are more likely to occur when high concentrations of EVs are present in a small volume with non-vesicular material; circumstances which are more likely within DC purification strategies rather than SEC purification of EVs [8]. However, EV aggregates may be overestimated by TEM and AFM methodology whereby surfaces capture EVs. Furthermore, vesicle morphology can be influenced by TEM and AFM methodology processes [6,51,52]. Both methods exhibited EV loss as demonstrated by the presence of EVs in the flow through (ESP), confirming that both purification methods are selective upon EV isolation.

When analysing the protein composition of purified EVs, it was apparent that SEC methods purified a greater protein yield within the EV samples (whole lysed, soluble protein fraction and insoluble protein fraction) when compared to DC preparations. These findings compare well with research identifying a greater number of particles to protein yield via SEC methods compared to DC methods and other commercial EV purification systems [2,14,15,53,54]. SEC has further been found to remove contaminating proteins from difficult sample types compared to DC such as albumin, cholesterol and apolipoprotein AI from blood plasma EVs and lipopolysaccharide binding protein from prokaryote EVs [14,55,56]. Conversely, Mol *et al*., [57] observed no significant differences in EV protein or particle yield between both purification methods. There is an inverse relationship between EV purity and the protein yield, where the lower the ratio the more impure the sample [1,10]. Therefore, sample purity may have consequent effects on experimental investigations [10]. Similar protein levels were observed between whole lysed EV and ESP samples purified by DC, yet the protein concentration of ESP was nearly threefold that of the whole lysed EV samples during SEC purification. This suggests again that SEC purification methods have an increased EV purity to protein yield ratio than DC based methods. This significant variation in ESP protein yield is most likely a direct result from sample wash steps involved with DC purification of EVs. Therefore, multiple wash steps are likely to increase EV loss and, as Webber and Clayton [10] acknowledged, are ineffective at removing protein contaminants, where there was only a 2-fold increase in particle to protein yield, compared to the crude pellet. Furthermore, there is the possibility that protein will sediment along with EVs during DC rather than be separated from EVs. Thus, in the current work SEC EV purification delivered a higher yield EV preparation in addition to higher yields of EV depleted ESP.

Whole lysed EV and ESP samples displayed different protein profiles as observed by one dimensional SDS-PAGE electrophoresis, suggesting that both EV purification methods isolate EVs from remaining *F*. *hepatica* secretions or ESP to varying degrees of purity. Following mass spectrometry analysis, we identified a greater total number of proteins associated with DC purified EVs than SEC purified EVs. However, SEC purified EV samples showed a greater diversity of EV functions in biological process, molecular function and cellular component gene ontology categories than DC purified EVs. The most common gene ontology terms identified in biological process, molecular function and cellular component were present in EVs from both purification methods. A comparable result was found investigating cell culture EVs, where fewer proteins (388 proteins) were identified in SEC purified EVs than in DC purified EVs (421 proteins), although fewer proteins (147 proteins) overlapped between SEC and DC EVs compared to the current study [53]. This result further suggests that DC purification alters EV protein composition to a greater extent than SEC purification, possibly isolating different EV subpopulations, as well as purifying greater amounts of contaminant. Thus, supporting SEC purification methods for an increased EV purity to protein yield ratio than DC based methods.

The most abundant proteins found in *F*. *hepatica* EVs, per rank, showed DC consistently had a greater amount of unique peptides hit per protein than SEC. Cwiklinski *et al*. [24] similarly analysed *F*. *hepatica* EV proteome where all 180 proteins identified were also found in the current investigation. However, the top unique peptide hits (where soluble and insoluble fraction data was combined) from Cwiklinski *et al*. [24] were not comparable to the current experimental results. Cwiklinski *et al*. [24] did not investigate a biological representation of the whole *F*. *hepatica* EV proteome, but instead EVs isolated from 120,000 *x* g ultracentrifugation spin only. In addition, a different mass spectrometry methodology and analysis was performed likely explaining DC EV differences. The top 50 proteins observed in EVs purified using SEC were also present in the DC EV purified proteome. However, three proteins were identified in the top 50 proteins of DC purified EVs, but were not present in the full SEC isolated EV proteome. Common EV markers were present in the top 50 EV proteins using both purification methods although tetraspanin was identified in the top 50 EV protein hits using SEC purification only and gelosin and phosphoglycerate kinase EV markers were observed in the top 50 protein hit using DC purification only. This may indicate that SEC and DC purification methods also purify different EV sub-populations, or that EV sub-populations are purified in differing quantities within each method.

Further investigation discovered that there was a 2.6 fold greater number of protein identifications in DC purified EVs only, compared to SEC purified proteins. Of these DC EV unique proteins many have been identified previously in ESP and tegument proteomic studies. With pathogens, the separation of EVs from additional pathogen components such as the platyhelminth tegument is vital to fully assess the role of the pathogen EV. The recognised increase of tegumental proteins found within the DC purified EVs further supports that SEC purified EVs have a greater EV purity to protein yield than DC purified EVs. Furthermore, current international EV purification methods report that chromatographic methods, including SEC, produce less contamination by non-vesicular proteins and macromolecule structures than ultracentrifugation, thus is likely to account for the reduction of tegumental proteins in SEC EVs [58].

Akin to the work of Benedikter *et al*. [53] gene enrichment analysis demonstrated comparable enriched gene ontology terms for both purification methods in biological process, molecular function and cellular component categories against the *F*. *hepatica* genome. Interestingly, only GO terms for translation biological process ribosome cellular component and structural constituent of the ribosome were significantly less enriched in DC purified EVs compared to SEC purified EVs, while DNA-templated regulation of transcription was enriched significantly in DC purified EVs compared to SEC purified EVs. This further suggests that DC and SEC purification methods isolate differing EV populations with altered functions. Cwiklinski *et al*. [24] previously discovered that EV sub-populations secreted from *F*. *hepatica*, contained different relative peptidase activities. A range of other studies have also identified that nucleic acids [59–64] and proteins [60,64–67] are selectively packed into EV subtypes. It has further been found that purification methodologies have differential affinity for protein EV markers and by extension for different EV sub-population using human urine samples [68]. This research further supports the current work in that DC and SEC purification methods isolated EVs of different function and different levels of purity.

An increased EV purity to protein yield ratio demonstrated in SEC purification over DC based methods was further reinforced by Western blotting revealing higher recognition by Anti-FhFABP V, Anti-FhGST-S1 and Anti-FhCat-L1 in the EV soluble fraction using DC purification rather than SEC. Only protein fragments from FABP III were identified within the mass spectrometry peptide analysis from the FABP protein family. Importantly, comparable levels of recognition by Anti-FhFABP V and Anti-FhGST-S1 were seen in DC and SEC ESP depleted of EVs, yet a greater recognition from Anti-FhCat-L1 was observed in SEC EV depleted ESP demonstrating increased separation of ESP from EVs in SEC methods. Baranyai *et al*. [55], also using Western blotting, demonstrated that SEC produced a greater EV purity to protein yield ratio, as higher albumin concentrations were seen in DC methods than SEC methods in mouse blood plasma. Correspondingly, vesicle markers have been found at greater concentrations in EV samples using SEC methods than DC [2]. Interestingly, there were a greater variety of cathepsin L protease isoforms identified in EVs purified by SEC, rather than EVs purified by DC. Of these proteins, there was a greater number of cathepsin L1A and cathepsin L1D, but a lower abundance of cathepsin L5 in SEC purified EVs, compared to DC purified EVs. Cathepsin L proteases have functional roles within immune evasion, nutrition and migration. In particular, cathepsin L1 and L2 proteases degrade host haemoglobin, immunoglobulin and interstitial matrix proteins such as fibronectin, laminin, and native collagen [69,70]. Different cathepsin L protease clades, have distinct substrate abilities, indicating that they have different roles in parasite biology. In the case of cathepsin L5, these roles are yet to still be determined [47,71]. In previous proteomic studies investigating the surface and membrane fractions of *F*. *hepatica* EVs [24], members of the CL1 clade (1A,1B and 1D) have all been identified to be membrane associated, yet members of CL5 have not. Therefore, the SEC approach identified more membrane associated cathepsin L in comparison to DC, identifying increased soluble cathepsin L derived from the EV contents or from contamination from ESP.

Interestingly, Anti-FhGST-S1 recognition was strongly observed in the insoluble EV fraction and a higher protein abundance of FhGST-S1 was suggested using SEC purification. Therefore, FhGST-S1 could potentially act as a novel marker for *F*. *hepatica* EVs given its identification in the insoluble EV fraction. The function of FhGST-S1 in *F*. *hepatica* includes prostaglandin synthesis which establishes host infection, egg development and embryogenesis, host immune modulation and potential triclabendazole drug response in phase III sequestration based detoxification [40]. Recent studies have found that parasite EVs can communicate with host cells therefore, the function FhGST-S1 in *F*. *hepatica* EVs could involve host infection establishment and host immune modulation [23,28,29,72–82]. However, further investigation upon this proposal is required to determine the role and location of FhGST-S1 in EVs. This finding indicates the importance of EV purification methodology upon accurately identifying the abundance of pathogen markers and vaccine candidates in EVs.

To claim that SEC should be used as a gold standard method for isolating EVs from parasite culture media, additional isolation methodologies (density gradient, sucrose cushion, precipitation methods and immunoaffinity isolation) will need to be compared with SEC methodology. It is acknowledged that within any purification method, many variables, such as the number of wash steps, will affect the purity and functionality of purified EVs. Given that all EV helminth studies to date have used DC purification for downstream analysis, the present study findings suggest a change in approach may be required with SEC providing a promising purification method for isolating EVs from *in vitro* helminth cultures for downstream functional analysis in terms of minimising non-EV contaminants.

There is a strong demand for an established uniform protocol for EV purification. However, instead of finding the ultimate ‘gold standard’ for EV purification, from this investigation it appears to be of more importance that EV purity is standardised, rather than the purification protocol. Possibly, rather than the helminth EV field aiming to isolate EVs using a ‘gold standard’ method, there should be a ‘gold standard’ of purity assessment of EV samples used for experimentation, dependent upon the sample type (e.g. plasma, urine, cell culture media and parasite culture media). EV specific markers are also likely to be important to identify EV sample purity. Specific EV markers would add value to the assessment of EV sample purity and thus could be species specific. This is especially pertinent given that current EV markers are based on mammalian work [83]. In the current study, differential abundance of protein families (CAT L clades and egg based proteins) were noted during proteomic analysis that could be utilised for *Fasciola* specific EV purity markers. This would improve the standardisation of protocols and the comparability of results from scientific research.

Thorough proteomic investigation on EV protein composition from EVs purified by DC and SEC methodology identified that SEC purified EVs contained proteins with more functional properties than DC purified EVs. However, in order to confirm increased EV functionality and the improved functional benefit of using SEC for EV purification compared to other methodologies, additional functional studies comparing the isolation methodologies by independent host-parasite interaction groups would be required. For example, SEC and DC purified EVs could be cultured with host cells *in vitro* and host cell transcriptome data could be compared between the two isolation conditions, following the methodology undertaken using DC purified *O*. *viverrini* EVs [76]. However, as the findings from the current study support that SEC purified EVs contained proteins with more functional properties than DC purified EVs, speculation upon the validity of other EV functional studies which have purified EVs from parasite culture media using DC methodology is necessary. Therefore, more comparative research is required to understand the influence of EV purification methodologies upon functional studies.

In summary, the current study has challenged whether the most accepted EV purification technique in helminth research is optimal for functional studies, in comparison to SEC methods. Our discoveries using *F*. *hepatica* as a pathogen case study, suggest that SEC purification has a higher EV purity to protein yield ratio than DC purified EVs evidenced by reduced contamination from ESP and tegumental components, whilst still maintaining EV morphological characteristics. Furthermore, gene ontology terminology proposed that DC and SEC purification methods isolate differential EV sub-populations. Given the demonstrated variation in purification methodologies and the importance of understanding the function of EVs for potential downstream studies and application development, the authors suggest that for EV functional assays the purification methodology used should be of importance when designing experiments. This research has highlighted SEC EV isolation as a potential key methodology for functional EV research.

## Supporting information

S1 File MASCOT (Matrix Science, v2.4.1) extracellular vesicle protein accession information for each band sample analysed by liquid Chromatography tandem mass spectrometry per replicate. (XLSX)

S2 File BLAST2GO (https://www.blast2go.com/) extracellular vesicle reported protein accession information and Level 3 gene ontology descriptions of protein identifications found in differential centrifugation and size exclusion chromatography purified extracellular vesicles. (XLSX)

S3 File MASCOT (Matrix Science, v2.4.1) unique and total peptide hits for extracellular vesicle protein accessions purified by differential centrifugation and size exclusion chromatography. (XLSX)

S4 File GOATOOLS in python software gene enrichment analysis of extracellular vesicles purified using differential centrifugation and size exclusion chromatography. (XLSX)

S5 File The abundance of differential centrifugation or size exclusion chromatography purified extracellular vesicle only protein accessions and their association with other *F. hepatica* ESP or parasite tegument proteomic studies. (XLSX)

S6 File **Level 3 Gene ontology descriptions of extracellular vesicle protein identifications purified using differential centrifugation and size exclusion chromatography, differential centrifugation extracellular vesicle samples only and size exclusion chromatography extracellular vesicle samples only in (a) biological process, (b) molecular function and (c) cellular component functional categories in graphical format**.
(PDF)

S7 File **Gene enrichment comparison of extracellular vesicles purified using differential centrifugation and size exclusion chromatography methodology within (a) biological process, (b) cellular component and (c) molecular function categories against the F. hepatica genome in graphical format**.
(PDF)
