# Supplementary material for: The importance of extracellular vesicle purification for downstream analysis: A comparison of differential centrifugation and size exclusion chromatography for helminth pathogens
Source: PLoS Negl Trop Dis. 2019 Feb 27;13(2):e0007191. doi: 10.1371/journal.pntd.0007191 (PMC6411213; doi:10.1371/journal.pntd.0007191)
Supplement: S6 File — Level 3 Gene ontology descriptions of extracellular vesicle protein identifications purified using differential centrifugation and size exclusion chromatography, differential centrifugation extracellular vesicle samples only and size exclusion chromatography extracellular vesicle samples only in (a) biological process, (b) molecular function and (c) cellular component functional categories in graphical format. (PDF) [file pntd.0007191.s006.pdf]

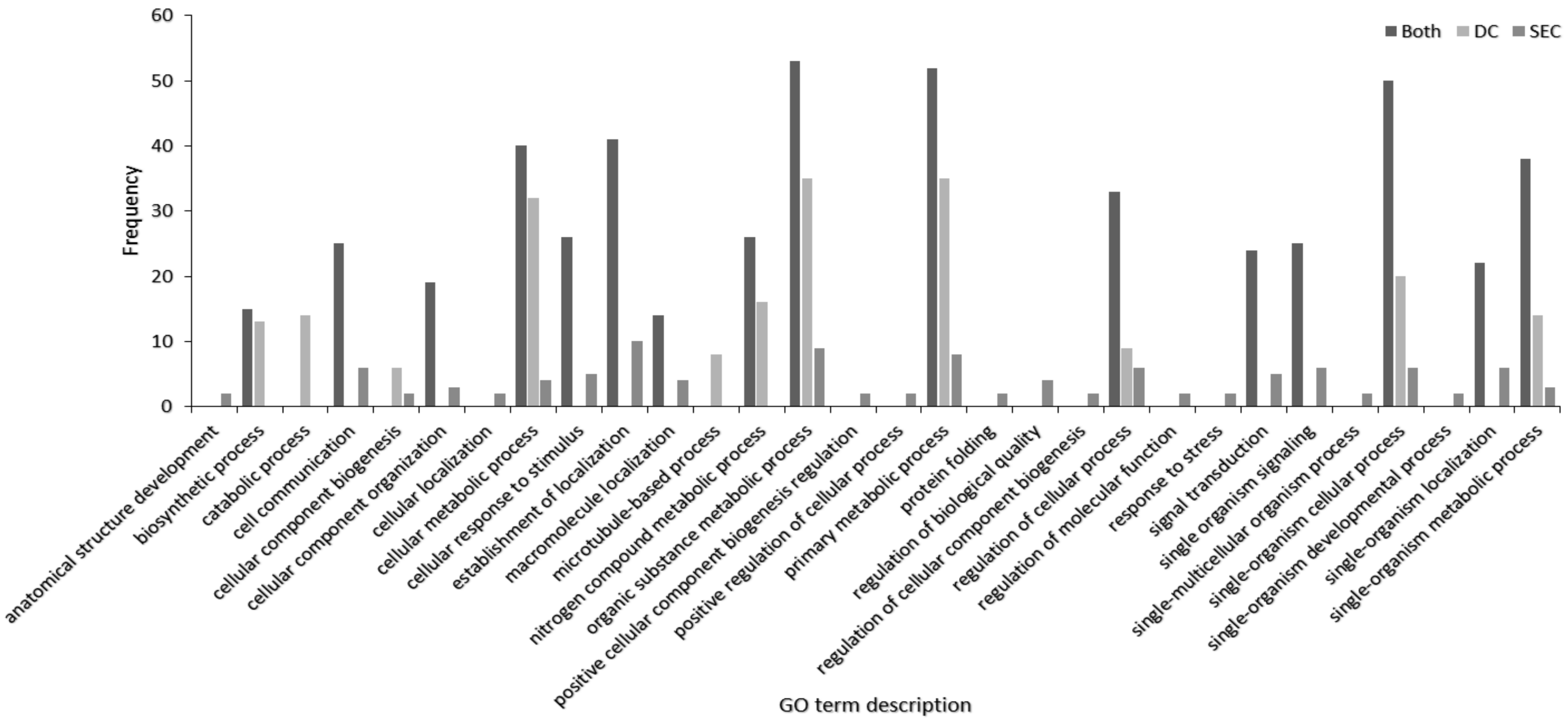

Level 3 Gene ontology descriptions of protein identifications observed in EVs common to both isolation methods, to DC EV samples only and to SEC EV samples only in biological process component functional categories. Data analysed using BLAST2GO (n= 2).

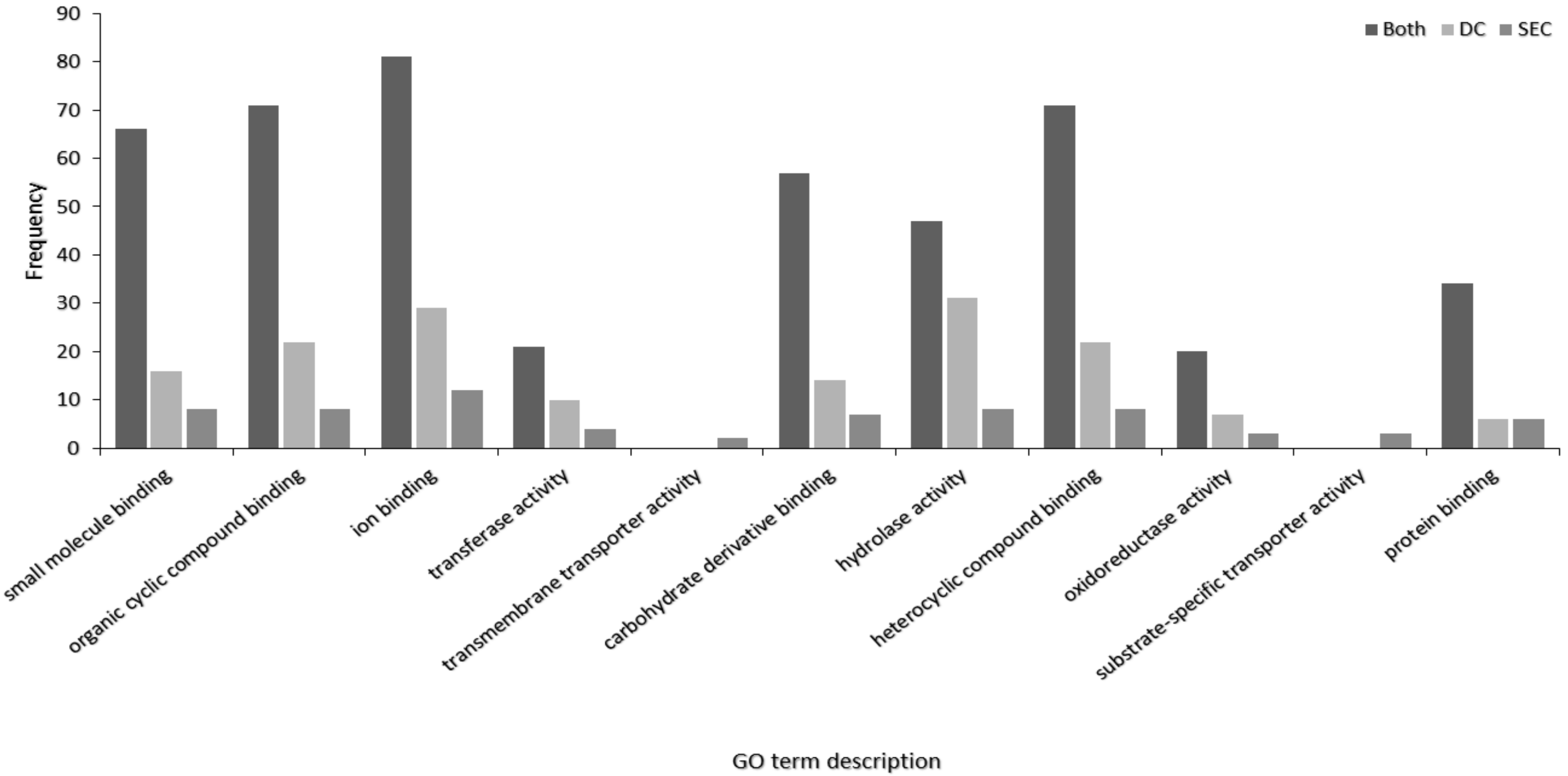

Level 3 Gene ontology descriptions of protein identifications observed in EVs common to both isolation methods, to DC EV samples only and to SEC EV samples only in molecular function functional categories. Data analysed using BLAST2GO (n= 2).

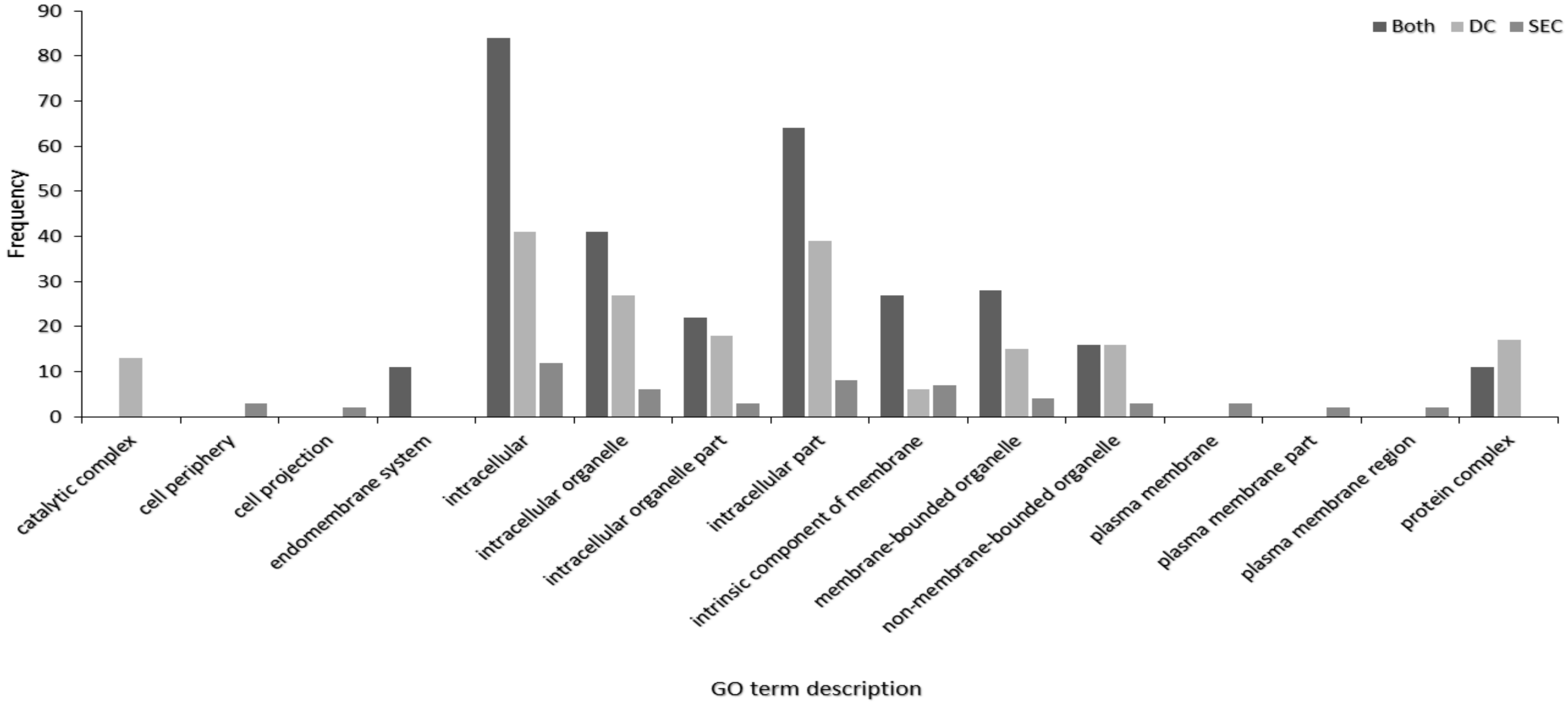

Level 3 Gene ontology descriptions of protein identifications observed in EVs common to both isolation methods, to DC EV samples only and to SEC EV samples only in cellular component functional categories. Data analysed using BLAST2GO (n= 2).
