# Supplementary material for: The importance of extracellular vesicle purification for downstream analysis: A comparison of differential centrifugation and size exclusion chromatography for helminth pathogens
Source: PLoS Negl Trop Dis. 2019 Feb 27;13(2):e0007191. doi: 10.1371/journal.pntd.0007191 (PMC6411213; doi:10.1371/journal.pntd.0007191)
Supplement: S7 File — Gene enrichment comparison of extracellular vesicles purified using differential centrifugation and size exclusion chromatography methodology within (a) biological process, (b) cellular component and (c) molecular function categories against the F. hepatica genome in graphical format. (PDF) [file pntd.0007191.s007.pdf]

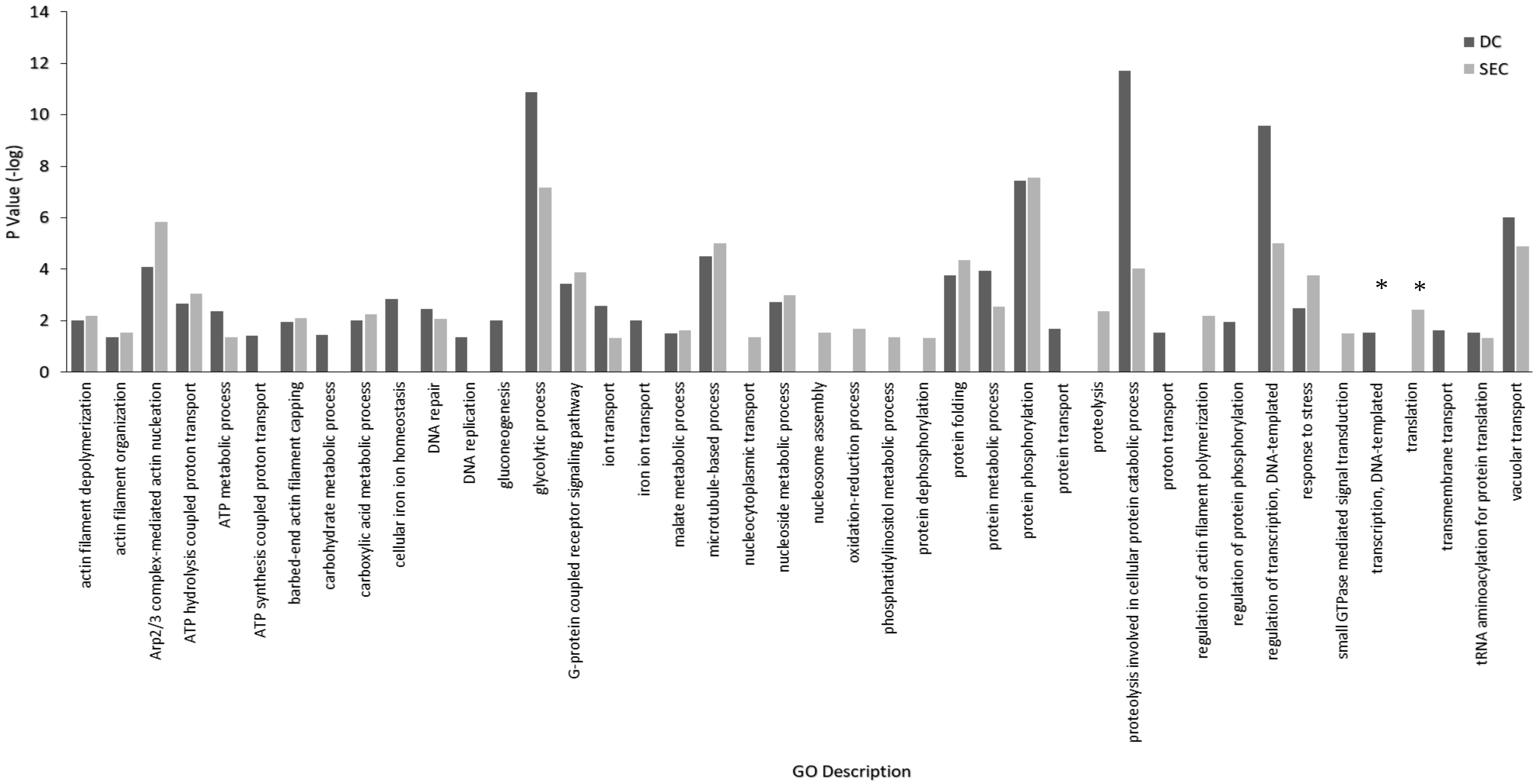

Gene enrichment analysis comparison between SEC and DC *F. hepatica* EV purification methods within biological process categories against the *F. hepatica* genome. Significant processes lower than 0.05 probability, presented using -log transformation were identified by asterisks. Data analysed using GOATOOLS in python software.

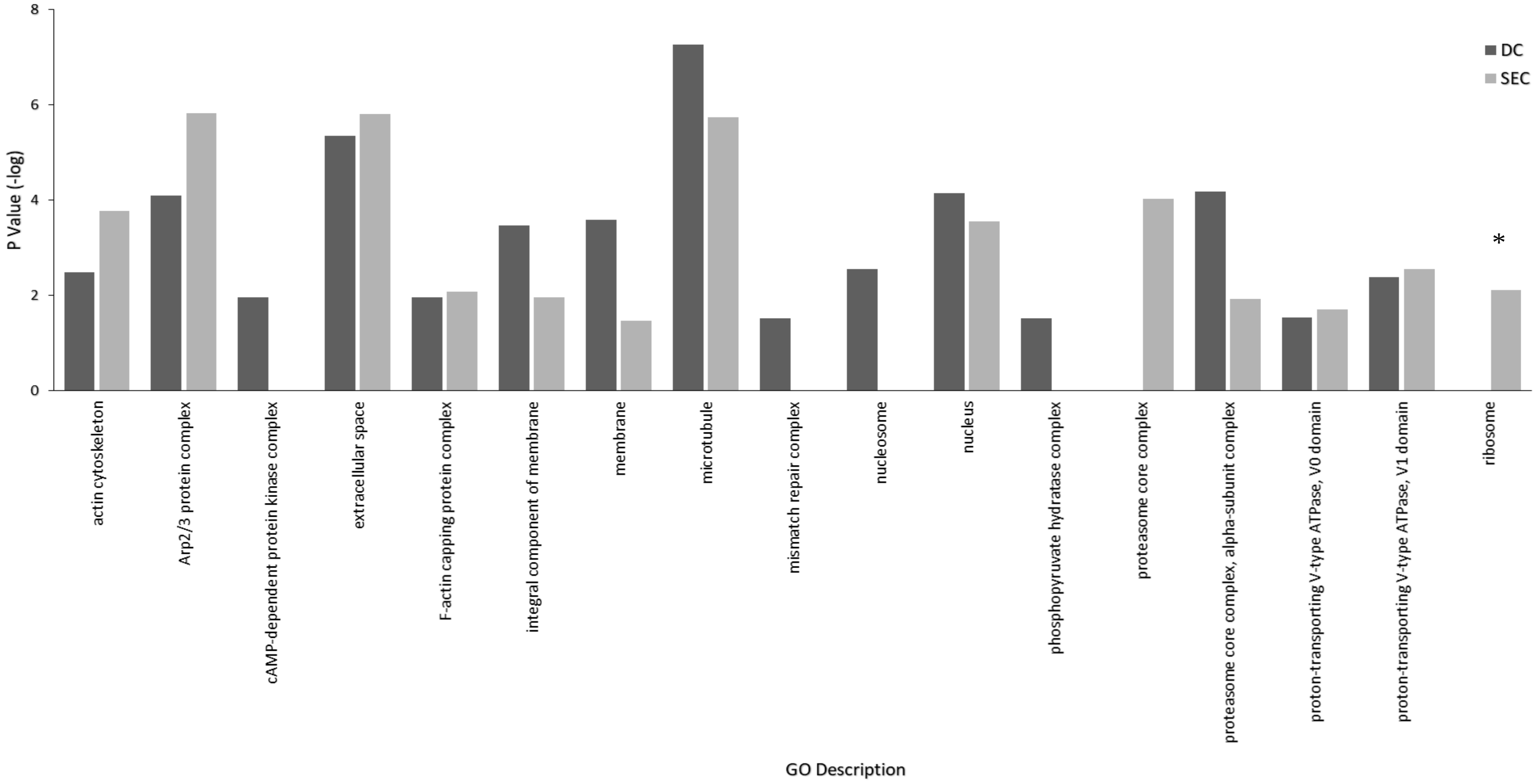

Gene enrichment analysis comparison between SEC and DC purification methods within cellular component categories against the *F. hepatica* genome. Significant processes lower than 0.05 probability, presented using -log transformation were identified by asterisks. Data analysed using GOATOOLS in python software.

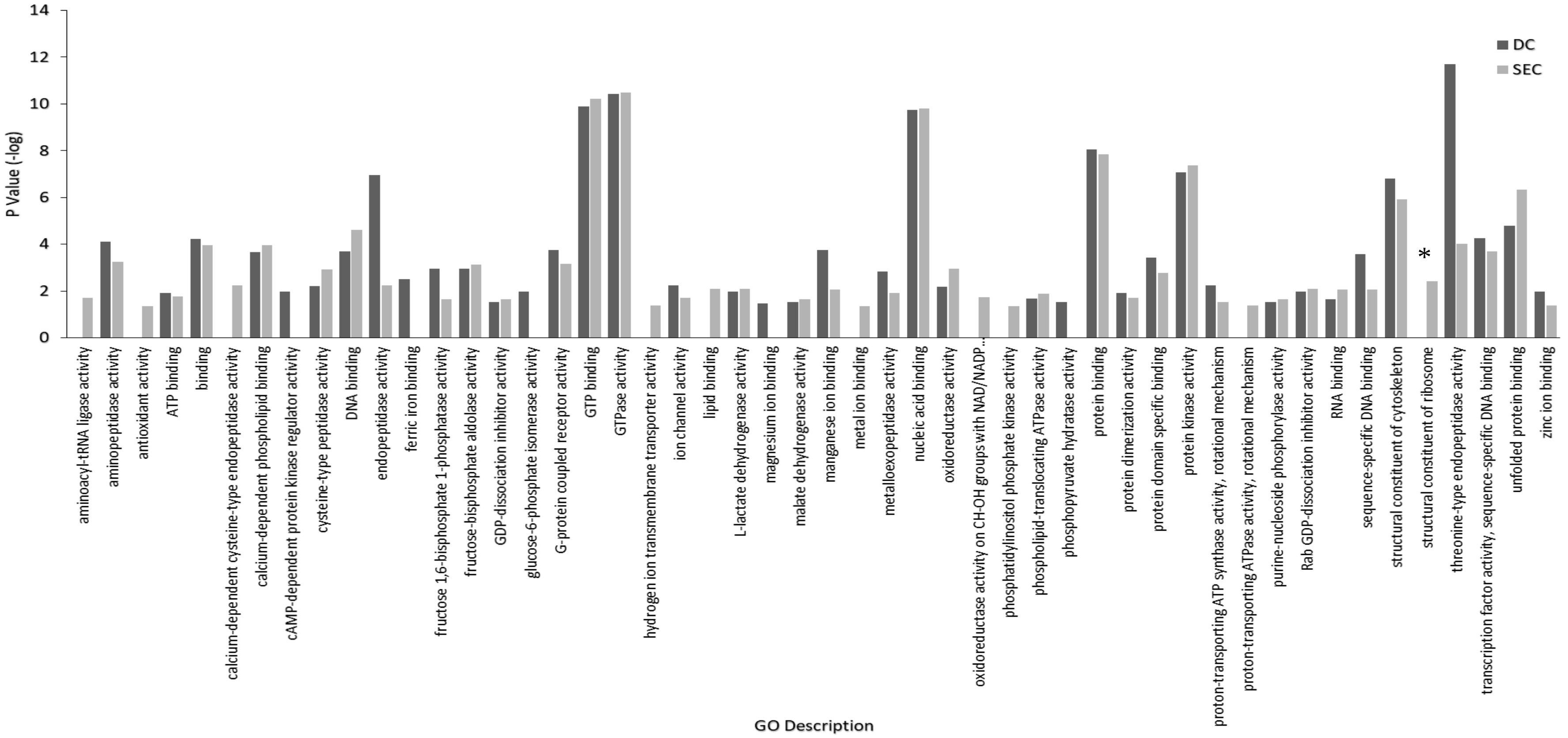

Gene enrichment analysis comparison between SEC and DC purification methods within molecular function categories against the *F. hepatica* genome. Significant processes lower than 0.05 probability, presented using -log transformation were identified by asterisks. Data analysed using GOATOOLS in python software.
